# Enteric Opportunistic Infection and the Impact of Antiretroviral Therapy among HIV/AIDS Patients from Tehran, Iran

**Published:** 2019-04

**Authors:** Hossein MASOUMI-ASL, Khadijeh KHANALIHA, Farah BOKHARAEI-SALIM, Abdoulreza ESTEGHAMATI, Saeed KALANTARI, Maryam HOSSEINYRAD

**Affiliations:** 1. Research Center of Pediatric Infectious Diseases, Institute of Immunology and Infectious Diseases, Iran University of Medical Sciences, Tehran, Iran; 2. Department of Virology, School of Medicine, Iran University of Medical Sciences, Tehran, Iran; 3. Department of Infectious Diseases, Rasoul-e-Akram Hospital, Iran University of Medical Sciences, Tehran, Iran; 4. Consult Center of Behavior Diseases, West Health Center, Iran University of Medical Sciences, Tehran, Iran

**Keywords:** Opportunistic infection, HIV, Coccidian, Microsporidian

## Abstract

**Background:**

Opportunistic parasites have been identified as human pathogens, especially in immunodeficient patients. Microsporidian and coccidian infections cause chronic diarrhea as common clinical manifestation in HIV positive patients. In this study, the frequency of opportunistic infections, including microsporidian and coccidian infections, was evaluated in HIV/AIDS patients from Tehran and phylogenic analysis was performed for *E. bieneusi* isolates from these patients.

**Methods:**

One hundred and two stool samples were collected from confirmed HIV/AIDS patients, referred to Consult Center of Behavior Diseases, West Health Center, Iran University of Medical Sciences in Tehran, Iran. The samples were transferred to Research Center of Pediatric Infectious Diseases, Institute of Immunology and Infectious Diseases, Iran University of Medical Sciences from Jan 2016 to Dec 2016. After conventional formalin-ether concentration, aniline blue staining method and acid-fast staining technique were used for detection of microsporidian spores and *Cyclospora* oocysts. DNA was extracted and nested PCR was performed.

**Results:**

Two (1.96%) cases were found to be positive for intestinal microsporidia infection using aniline blue staining method and were confirmed as *E. bieneusi* by nested PCR. One patient was found with *Cyclospora cayetanensis* infection by acid-fast staining method and PCR. *Giardia lamblia* and *Blastocystis hominis* were detected as non-opportunistic parasites in 1/102 (0.98%) and 2/102 (1.96%) of the HIV positive patients, respectively.

**Conclusion:**

With respect to the use of antiretroviral therapy (ART) in HIV positive patients, we found a low frequency of infection.

## Introduction

Opportunistic parasites including microsporidian and coccidian infections have been identified as human pathogens, especially in immunodeficient individuals like organ transplant recipients (1), cancer patients (2), and HIV/AIDS patients (3). However, there are some reports of microsporidial infection in immunocompetent people also, especially those like travelers, children, and healthy human population from all over the world (4, 5).

The most common microsporidial infections are *Enterocytozoon bieneusi* and *Encephalitozoon intestinalis* in humans (6). *E. bieneusi* is a zoonotic pathogen that infects animals and causes infection in humans (7, 8). *E. bieneusi* infection causes chronic diarrhea and biliary illness and it is has been reported in individuals infected with HIV (4). Prevalence rate of *E. bieneusi* has been reported between 2.5%–51% for HIV-seropositive adult patients with diarrhea (9–11) and 4.6% for patients without diarrhea (12).

Diagnosis of microsporidia is based on the identification of spores by staining methods like chromotrope 2R, aniline blue and calcofluor white (13, 14), and various molecular methods (3, 12, 15).

*Cyclospora cayetanensis* infection has recently emerged as one of the opportunistic infections with worldwide distribution. A number of outbreaks have been reported in the United States and Canada (16). The oocyst of *C. cayetanensis has* been identified in the feces of immunocompetent people who travel to developing countries or consume contaminated fruits or salads, and in patients with AIDS. In Pune, India, *Cyclospora* was reported in 0.7%–3.3% of HIV positive individuals with diarrhea (17).

Oocysts of *Cyclospora* can be distinguished by direct examination of wet smear. They can also be observed by acid-fast staining method and diagnosis of oocysts sporulation after preservation in dichromate potassium (2.5%) can be made (16, 18).

This study aimed to evaluate the frequency of opportunistic infections, including microsporidian and coccidian infections, among HIV/AIDS patients in Tehran.

## Methods

In this cross-sectional study, 102 stool samples were collected from confirmed HIV/AIDS patients, with or without diarrhea, referred to Consult Center of Behavior Diseases, West Health Center, Iran University of Medical Sciences, Tehran, Iran. The stool samples were transferred to Research Center of Pediatric Infectious Diseases, Institute of Immunology and Infectious Diseases, Iran University of Medical Sciences, from Jan 2016 to Dec 2016.

Wet mount smear was prepared from concentrated samples and all samples were observed under light microscope. Acid-fast staining was carried out for all samples and slides were observed and evaluated for detecting *Cyclospora* and another coccidian like cryptosporidium oocysts.

### Ethical issues

This study was approved by the Ethics Committee of Iran University of Medical Sciences in accordance with the Helsinki Declaration and guidelines and all human participation has been obtained in accordance with informed consent.

### Cyclospora

The stool was evaluated by direct examination of wet smear and conventional formalin-ether concentration technique was performed, smear was prepared and fixed with methanol. Next, the slide was stained with modified acid-fast staining method (18). The stool was washed in Phosphate-buffered saline (PBS) and preserved in dichromate potassium (2.5%) for following up oocyst sporulation, and finally, autofluorescence of oocyst was observed with immunofluorescence microscope for further confirmation.

DNA extraction was performed by (Roche Diagnostics GmbH, Mannheim, Germany) according to the manufacturer’s instructions, with some modifications.

All primers used for *C. cayetanensis* were used from previously defined regions of the 18S ribosomal RNA gene in *C. cayetanensis.* PCR was performed in a 25 μL mixture containing the template (3 μL of DNA), 2.5 U of Taq DNA polymerase, 2.5 μL of 10x PCR buffer, 20 pmol of each primer, 100 μmol dNTPs, and 0.15 mmol MgCl_2_.

Nested PCR was performed by initial denaturation of 5 min at 95 °C, followed by a cycling program consisting of 35 cycles of denaturation at 94 °C for 30 sec, annealing at 53 °C for 30 sec, and extension at 72 °C for 90 sec. A final extension at 72 °C for 10 min was followed. A 636-bp product was expected after this round of PCR. The second round was similar to the first one, with the exception that the annealing temperature was 60 °C. The inner primers were expected to produce a 294-bp product in case of the presence of *C. cayetanensis* DNA as the template.

Nested PCR for the detection of *Cyclospora* was performed using the outer primers F1E (5′-TACCCAATGAAAACAGTTT-3′) and R2B (5′-CAGGAGAAGCCAAGGTAGG-3′). The inner primers used were F3E (5′-CCTTCCGCGCTTCGCTGCGT-3′) and R4B (5′-CGTCTTCAAACCCCCTACTG-3′) (15).

### E. bieneusi

Formalin-ether concentration method was performed and after 5 min of methanol fixation, aniline blue staining was done for the detection of microsporidial spores (13).

DNA extraction was performed by (Roche Diagnostics GmbH, Mannheim, Germany) according to the manufacturer’s instructions and nested PCR was performed using *E. bieneusi* specific primers, designed based on the small subunit (SSU) of rRNA gene.

The nested PCR was done using a set of primers that were specific for *E. bieneusi* ITS region as well as a portion of the flanking large and small subunit ribosomal RNA genes (400 bp). The outer primers were EBITS3 (5′-GGTCATAGGGATGAAGAG-3′) and EBITS4 (5′-TTCGAGTTCTTTCGCGCTC-3′), and the inner primers were EBITS1 (5′-GCTCTGAATATCTATGGCT-3′) and EBITS2.4 (5′-ATCGCCGACGGATCCAAGTG-3′). Finally, these reactions produced a fragment of 389 bp (12).

The reaction mixture (25 μL) contained 0.15 mM MgCl2, 2.5 μL of 10x PCR buffer, 100 μM dNTPs, 20 pmol of each primer, and 2.5 U of Taq DNA polymerase. PCR was performed as follows: denaturation at 94 °C for 3 min, followed by 35 cycles of amplification (denaturation at 94 °C for 30 sec, annealing at 57 °C for 30 sec, and elongation at 72 °C for 40 sec), and a final extension at 72 °C for 10 min. Conditions for the secondary PCR were identical to that of the primary PCR, except that only 30 cycles were carried out at an annealing temperature of 55 °C. These reactions produced fragments of 435 and 389 bp, respectively (12).

Finally, PCR products were electrophoresed on 2% agarose gel. Negative and positive controls were included in all sets of PCRs.

### Sequencing

The second round of PCR was performed with inner primers and PCR products were purified using the High Pure PCR Product Purification Kit (Roche Diagnostic, Mannheim, Germany) according to the manufacturer’s instructions and were used for direct sequencing using the dye termination method and an ABI 3730xl sequencer (19).

## Results

Out of 102 HIV positive patients, 70 (68.6%) were males and 32 (31.4%) were females including 9 patients with CD4 T-cell count< 200 cells /μl, 17 patients with CD4 200–500 cells/μl, and 76 patients with CD4 > 500 cells/μl. The mean age of patients was 31 yr (range of 19–48 yr). Among 102 stool samples, two (1.96%) cases were found to be positive for intestinal microsporidia infection using aniline blue staining method and were confirmed as *E. bieneusi* by nested PCR. One patient (0.98%) was found with *C. cayetanensis* infection by acid-fast staining method and PCR. *G. lamblia* and *B. hominis* were detected as non-opportunistic parasites in 1/102 (0.98%) and 2/102 (1.96%) of the HIV positive patients, respectively (Table 1).

**Table 1:** Enteric opportunistic and non-opportunistic parasites among HIV/AIDS patients

***Parasites***	***CD4 > 500 cells/μl N=76***	***CD4 200–500 cells/μl N=17***	***CD4 < 200 cells/μl N=9***	***Total N (%)***
*E. bieneusi*			2	2(1.96)
*Cyclospora cayetanensis*			1	1(0.98)
*Giardia lamblia*	1			1(0.98)
*Blastocystis hominis*	1	1		2(1.96)
Total	2(1.96%)	1(0.98%)	3(2.9%)	6/102(5.9)

**Fig. 1:**
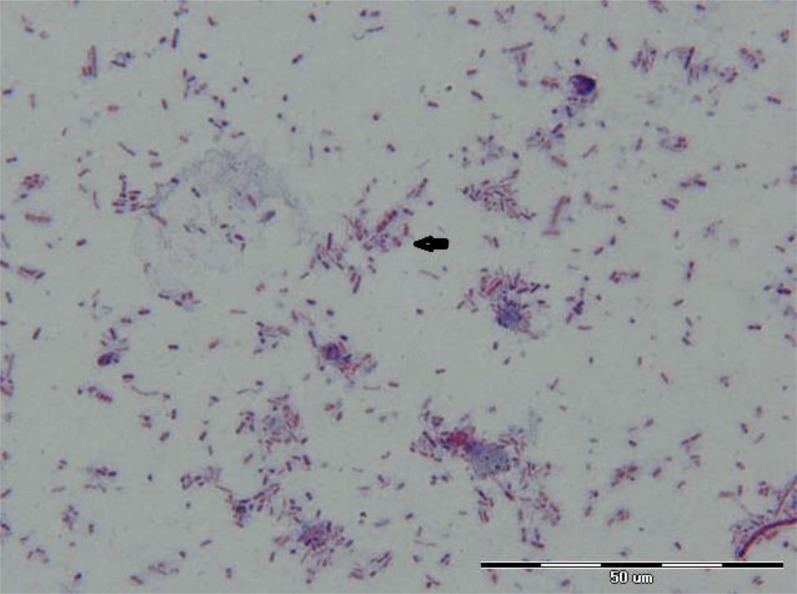
Appearance of *E. bieneusi* spores in stool samples collected from HIV positive Patients by aniline blue staining method, 1000x magnification (origin picture)

**Fig. 2:**
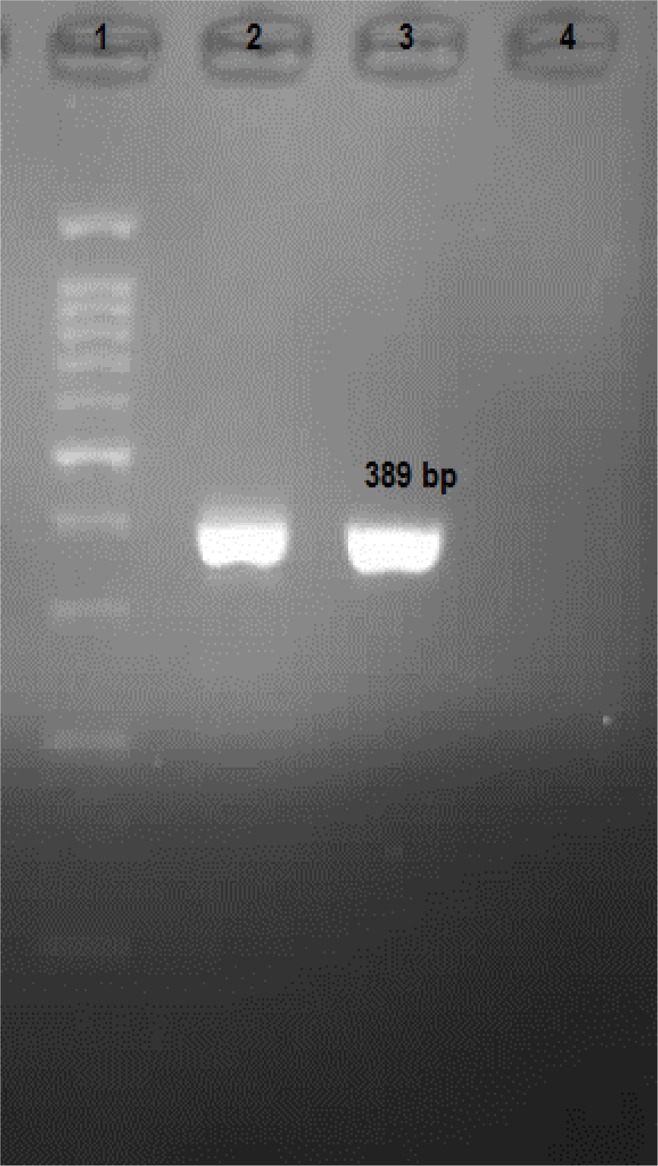
Agarose gel Electrophoresis of PCR products of E*. bieneusi in* Iranian HIV positive patients.1:100 bp DNA ladder marker; 2–3 Positive E*. bieneusi* patients (389 bp); 4: Negative Samples

Microsporidian spores with aniline blue staining were oval and measured 1–1.5 μm in size (Fig. 1). The positive samples showed positive results for nested PCR for *E. bieneusi* also. A band of 389 bp corresponding to *E. bieneusi* was observed in positive cases (Fig. 2). These two positive *E. bieneusi* patients had chronic diarrhea as main clinical manifestation and showed a CD4 count of less than 200 cells/μl.

The result of sequencing of two *E. bieneusi* positive patients in this study based on SSU rRNA gene ITS region demonstrated 99% identity with *E. bieneusi* isolates HNSC9, 178, HNZM19 and Chan L46, and 98% identity with isolates HNZM54, 356, and 601.

**Fig. 3:**
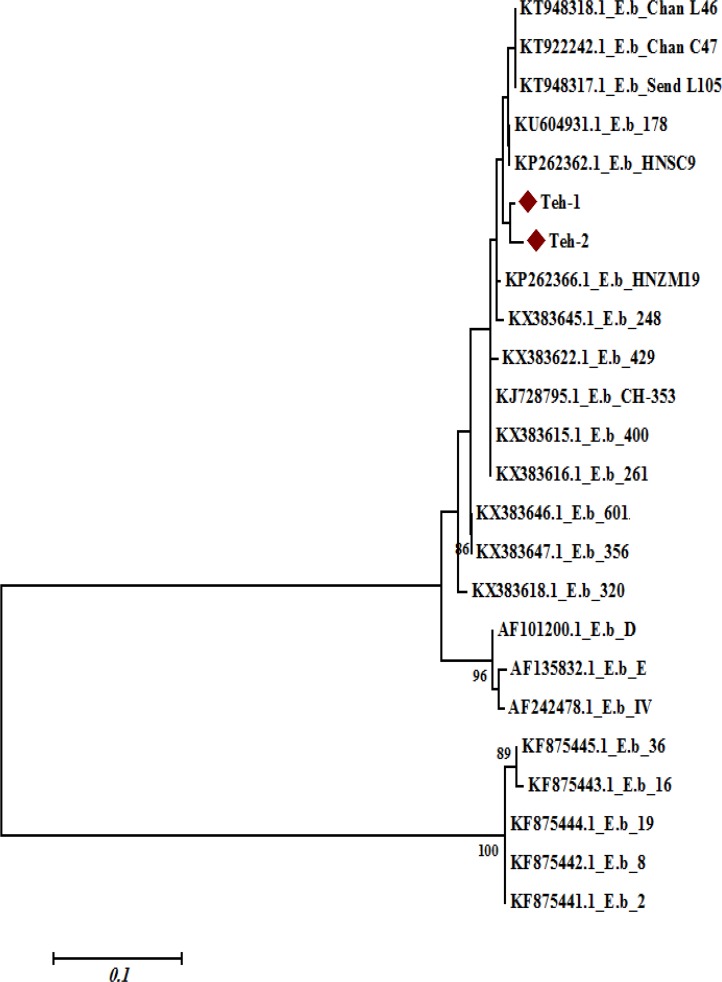
Phylogenetic tree based on E*. bieneusi* nucleotide sequences from two individuals with E*. bieneusi* infection and those corresponding to different E*. bieneusi* genotypes taken from the GenBank database. Bootstrap values ≥70 achieved after 1000 replicates are shown at the nodes

The sequences were deposited in GenBank with accession numbers MF163429 and MF163430 (Fig. 3).

**Fig. 4:**
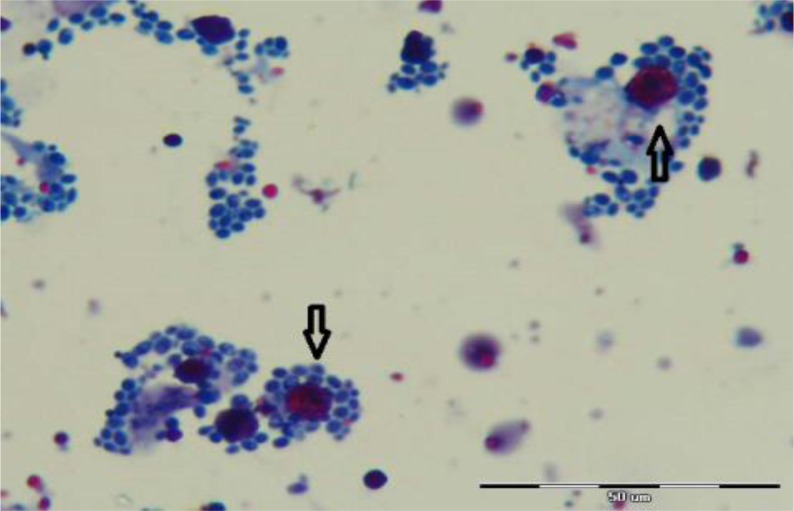
Appearance of *C. cayetanensis* oocysts in Stool samples collected from HIV positive patients by modified acid fast staining method, 1000x magnification (origin picture)

*C. cayetanensis* oocysts were found in a HIV positive patient with watery diarrhea and CD4 count less than 200 cells/μl. The oocysts were almost 8–10 μm in diameter and partial acid-fast positive with wrinkled edge in some oocyst cases (Fig. 4). The result of preservation in dichromate potassium (2.5%) and oocysts sporulation showed that oocysts had two sporocysts and each sporocyst contained two sporozoites.

For further confirmation, autofluorescence of oocyst was observed under immunofluorescence microscope.

**Fig. 5:**
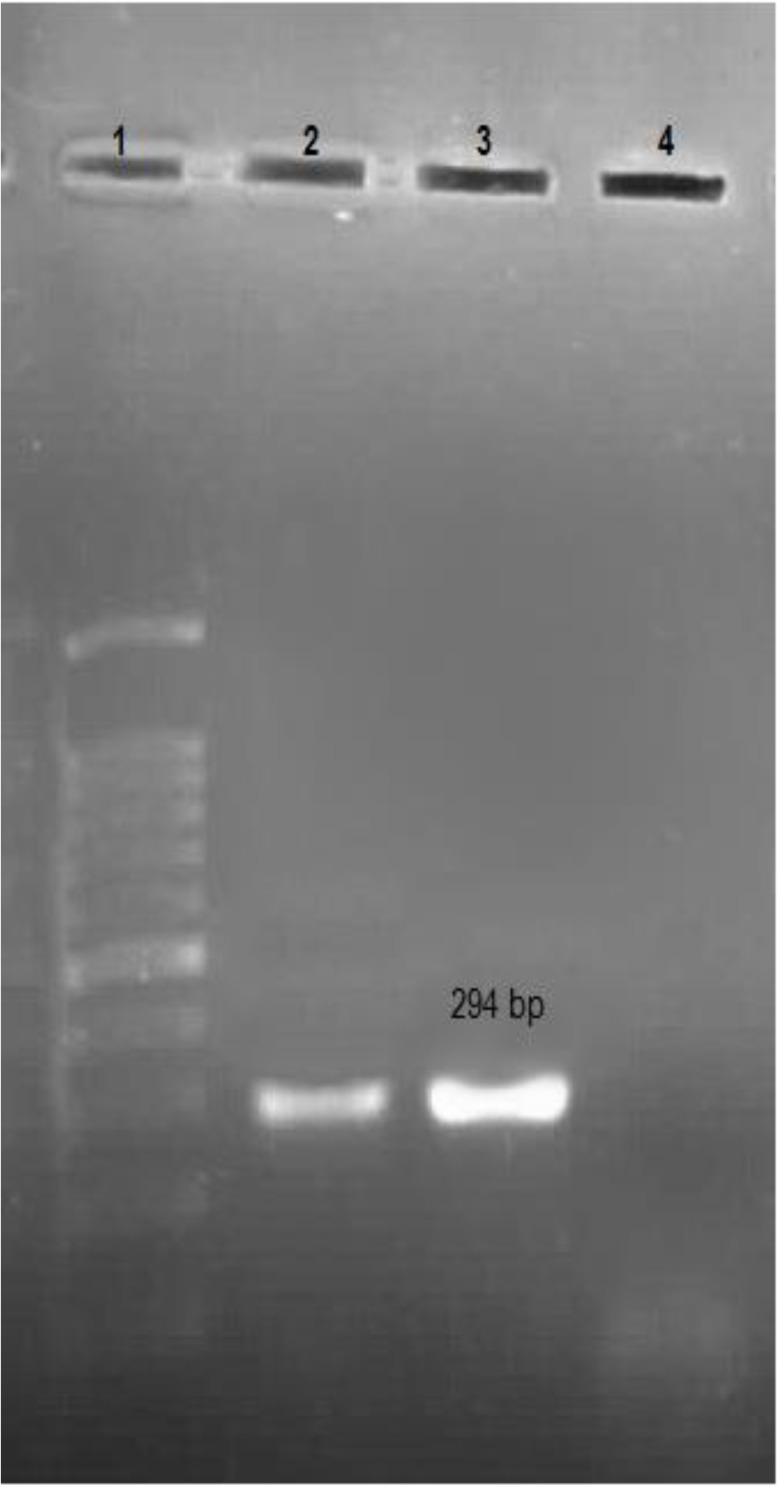
Agarose Gel Electrophoresis of PCR products of *C. cayetanensis in* Iranian HIV positive patient. 1:100 bp DNA ladder marker; 2: Positive *C. cayetanensis* Patient; 3: Positive control (294 bp). 4: Negative sample

*C. cayetanensis* was also confirmed by nested PCR. A band of 294 bp corresponding to *Cyclospora* was observed in positive cases (Fig. 5). The result of sequencing and blast showed 99% identity with *C. cayetanensis* isolates H6 (1–4), PC1, and HMCCPR2. The result of 18S ribosomal rRNA sequences of *C. cayetanensis* described in this paper was deposited in GenBank with the accession number KY769936.

*G. lamblia* and *B. hominis* were detected as non-opportunistic parasites in 1/102 (0.98%) and 2/102 (1.96%) HIV positive patients with CD4 cell count more than 200 cells/μl in this study.

## Discussion

Opportunistic infections are found frequently in HIV positive patients, especially when the CD4 T-cell count is less than 200 cells/μl (3).

Microsporidiosis in HIV positive patients have been reported from Southeast Asia (India, Thailand), the Middle-East (Turkey), Europe, Africa (Tunisia, Mali, Uganda, Senegal, Zimbabwe), and Latin America (Brazil, Peru) (20).

Diagnosis of *E. bieneusi* infection is based on staining methods and visualization by light microscopy and electron microscopy, and PCR methods. The comparison of results obtained is difficult because of the differences between these methods (21). Prevalence of *E. bieneusi* in HIV positive patients with diarrhea in developed countries like Europe, Australia, and North America has been reported to be between 2%–78% (21–23), however, this rate was reported from 1.4%–4.3% in HIV positive patients without diarrhea (24).

In this study, 102 HIV positive patients were evaluated to assess the occurrence of protozoa opportunistic infection and two cases were shown to be *E. bieneusi* positive by staining and nested PCR. Both of these patients had chronic diarrhea. Sequencing confirmed the isolates as *E. bieneusi,* with 99% identity to *E. bieneusi* isolates HNSC9, 178, HNZM19, and Chan L46; and 98% identity with isolates HNZM54, 356, and 601.

Prevalence of enteric *E. bieneusi* in HIV positive patients has been reported in various countries, like Russia (1.2%) (25), Thailand (5.6%) (26), Vietnam (7.1%) (27), Nigeria (2.6%) (28), Switzerland (12.7%) in patients with diarrhea and 0.4% in patients without diarrhea (29), Holland (7.7%) (30), UK (8.3%) (31), Peru (3.9%) (32) using fecal samples by PCR method.

In a study, from 356 HIV positive patients, eight (2.2%) cases were identified as *E. bieneusi* by staining method and PCR in Shiraz (3).

In a study conducted in North India, *E. bieneusi* was reported in 2.5% of the HIV-infected individuals. Furthermore, both *C. cayetanensis* and *B. hominis* were detected in 3.3% of the patients (9).

A systematic review and meta-analysis had reported the overall prevalence of microsporidia infection in immunocompromised patients to be 8.18% in Iran. In addition, prevalence of microsporidia infection was 15.4% in immunocompromised patients with chronic diarrhea, 4.1% in patients without diarrhea, and 12.9% in patients with CD4 less than 200 cells/μl (33).

Some studies carried out in developed countries demonstrated that the prevalence of *E. bieneusi* in HIV positive patients is decreasing with the use of highly active antiretroviral therapy (HAART) (29, 34).

Prevalence of intestinal parasites in pre-ART and patients with ART treatment was 39% and 17.6%, respectively. All positive cases related to *Cryptosporidium spp* were found in the pre-ART group and significantly related to CD4 <200cells/μL (35).

Prevalence of intestinal parasites in HIV/AIDS positive patients was compared between pre-HAART and HAART groups in Brazil. Frequency of *Isospora belli, Cryptosporidium* sp. and *G. lamblia* were (4.8% and 1%), (8.1% and 0) and (7.9% and 1%) in pre-HAART and HAART treatment respectively that may due to effect of HAART treatment on intestinal parasites (36). Effect of antiretroviral therapy on opportunistic infection like cryptosporidiosis and microsporidiosis in HIV positive patients were evaluated. The result confirmed combination antiretroviral therapy is able to improve the course of cryptosporidiosis and microsporidiosis in HIV-1 positive patients (37). HAART treatment in HIV positive patients in Australia has led to a decrease in microsporidial infections from 11% in 1995 to 0% in 2004 (38).

Contrary to this, prevalence of intestinal parasites remained high in pre-ART in comparision with on-ART pateints 84.6% and 82.3% respectively. Opportunistic infection like *C. parvum*, *Microsporidium* spp. and *I. belli* were associated with CD4 <200cells/μL in on-ART (39).

HIV/AIDS patients referred to Consult Center of Behavior Diseases, West Health Center, Iran University of Medical Sciences in Tehran, were used for the analysis. Two cases of *E. bieneusi* among 102 HIV positive patients were found with chronic diarrhea as main clinical manifestation. Furthermore, low prevalence of E*. bieneusi* in this study was interpreted by administration of ART treatment in all patients and use sulfamethoxazole-trimethoprim treatment during the course of AIDS for patients with CD4 T-cell count less than 200 cells/μl.

*C. cayetanensis* is a coccidian protozoan observed in many different countries. There are some reports about presence of *C. cayetanensis* in HIV positive patients from Argentina (40), Turkey (41), and Iran (18). Prevalence of *C. cayetanensis* has been reported to be 3% in Cuba (42), 11% in Haiti (43), between 0.7%–3.3% in India (9, 17), 2.2% in Thailand (44), and 1% in Tanzania (45). In this study, one *C. cayetanensis* positive sample was detected in 102 stool samples collected from HIV positive patients. The sequencing of *C. cayetanensis* and blast alignment showed 99% identity with *C. cayetanensis* isolates H6 (1–4), PC1, and HMCCPR2.

No *Cryptosporidium* spp. was found from 102 stool samples collected from HIV positive patients by modified acid-fast staining method and *G. lamblia* and *B. hominis* as non-opportunistic parasites were detected in 1/102 (0.98%), and 2/102 (1.96%) HIV positive patients with CD4 T-cell count more than 200 cells/μl.

Some studies have reported a prevalence range from (1.5%–9.4%) of enteric coccidian, *G. lamblia* with prevalence range (3.1%–7.3%) and *B. hominis* (4.4%) as most common non-opportunistic parasites in HIV/AIDS patients in Iran (46, 47).

In this study, *E. bieneusi* and *C. cayetanensis* cases were detected from HIV positive patients as opportunistic infection. Phylogenetic mapping indicated that these Iranian *E. bieneusi* isolates were most closely related to *E. bieneusi* isolates HNSC9, 178, HNZM19, and Chan L46; and *C. cayetanensis* isolates showed identity with *C. cayetanensis* isolates H6 (1–4), PC1, and HMCCPR2.

## Conclusion

In this study, with the use of ART treatment in HIV positive patients and administration of sulfamethoxazole-trimethoprim treatment during the course of AIDS for patients with CD4 T-cell counts less than 200 cells/μl, frequency of microsporidian and coccidian infection was low.

## Ethical considerations

This study was approved by the Ethics Committee of Iran University of Medical Sciences code number (IR.IUMS.REC1394-01-131-25831). Ethical issues (Including plagiarism, informed consent, misconduct, data fabrication and/or falsification, double publication and/or submission, redundancy, etc.) have been completely observed by the authors.

## References

[1] Agholi M Hatam GR Motazedian MH (2013). HIV/AIDS-associated opportunistic protozoal diarrhea. AIDS Res Hum Retroviruses, 29:35–41. 22873400 10.1089/aid.2012.0119 PMC3537293

[2] Agholi M Hatam GR Motazedian MH (2013). Microsporidia and coccidia as causes of persistence diarrhea among liver transplant children: incidence rate and species/genotypes. Pediatr Infect Dis J, 32(2):185–187. 22982981 10.1097/INF.0b013e318273d95f

[3] Anane S Attouchi H (2010). Microsporidiosis: epidemiology, clinical data and therapy. Gastroenterol Clin Biol, 34(8–9):450–464. 20702053 10.1016/j.gcb.2010.07.003

[4] Bachur TPR Vale JM Coêlho ICB (2008). Enteric parasitic infections in HIV/AIDS patients before and after the highly active antiretroviral therapy. Braz J Infect Dis, 12(2):115–122. 18641847 10.1590/s1413-86702008000200004

[5] Cegielski JP Ortega YR McKee S Madden JF (1999). Cryptosporidium, Enterocytozoon, and Cyclospora infections in pediatric and adult patients with diarrhea in Tanzania. Clin Infect Dis, 28(2):314–321. 10064250 10.1086/515131

[6] Chawla R Ichhpujani R (2011). Enteric spore-forming opportunistic parasites in HIV/AIDS. Trop Parasitol, 1(1):15–19. 23507985 10.4103/2229-5070.72112 PMC3593466

[7] Conteas CN Berlin O LaRiviere MJ (1998). Examination of the prevalence and seasonal variation of intestinal microsporidiosis in the stools of persons with chronic diarrhea and human immunodeficiency virus infection. Am J Trop Med Hyg, 58:559–561. 9598441 10.4269/ajtmh.1998.58.559

[8] Coyle CM Wittner M Kotler DP (1996). Prevalence of microsporidiosis due to Enterocytozoon bieneusi and Encephalitozoon (Septata) intestinalis among patients with AIDS-related diarrhea: determination by polymerase chain reaction to the microsporidian small-subunit rRNA gene. Clin Infect Dis, 23:1002–1006. 8922793 10.1093/clinids/23.5.1002

[9] Daryani A Sharif M Meigouni M (2009). Prevalence of intestinal parasites and profile of CD4+ counts in HIV+/AIDS people in north of Iran, 2007–2008. Pak J Biol Sci, 12(18):1277–1281. 20384282 10.3923/pjbs.2009.1277.1281

[10] Dengjel B Zahler M Hermanns W (2001). Zoonotic potential of Enterocytozoon bieneusi. J Clin Microbiol, 39(12):4495–4499. 11724868 10.1128/JCM.39.12.4495-4499.2001 PMC88572

[11] Escobedo AA Núñez FA (1999). Prevalence of intestinal parasites in Cuban acquired immunodeficiency syndrome (AIDS) patients. Acta Trop, 72(1):125–130. 9924968 10.1016/s0001-706x(98)00091-6

[12] Espern A Morio F Miegeville M (2007). Molecular study of microsporidiosis due to Enterocytozoon bieneusi and Encephalitozoon intestinalis among human immunodeficiency virus-infected patients from two geographical areas: Niamey, Niger, and Hanoi, Vietnam. J Clin Microbiol, 45(9):2999–3002. 17634305 10.1128/JCM.00684-07 PMC2045311

[13] Field A Hing M Milliken S Marriott D (1993). Microsporidia in the small intestine of HIV-infected patients. A new diagnostic technique and a new species. Med J Aust, 158(6):390–394. 7683076

[14] Garcia LS Bruckner DA (1993). Diagnostic medical parasitology. Washington, DC:131–135.

[15] Ghaderipour M Khanaliha K Mohebali M (2017). Emerging Intestinal Microsporidia Infection in General Population in Jiroft District, Southeastern Iran: A Cross-sectional Study in 2013–2014. Iran J Public Health, 46(12):1697–1703. 29259945 PMC5734970

[16] Ghoyounchi R Ahmadpour E Spotin A (2017). Microsporidiosis in Iran: A systematic review and meta-analysis. Asian Pac J Trop Med, 10(4):341–350. 28552104 10.1016/j.apjtm.2017.03.017

[17] Khanaliha K Masoumi-Asl H Bokharaei-Salim F (2017). Double-stranded RNA viral infection of Trichomonas vaginalis (TVV1) in Iranian isolates. Microb Pathog, 109:56–60. 28478201 10.1016/j.micpath.2017.04.032

[18] Khanaliha K Mirjalali H Mohebali M Tarighi F Rezaeian M (2014). Comparison of three staining methods for the detection of intestinal Microspora spp. Iran J Parasitol, 9(4): 445–451. 25759724 PMC4345082

[19] Khanaliha K Mohebali M Davoudi S (2015). Detection of emergence Cyclospora cayetanensis in A HIV+/AIDS patient with diarrhea from Tehran: a case report. Iran J Public Health, 44(6): 865–868. 26258100 PMC4524312

[20] Kulkarni SV Kairon R Sane SS (2009). Opportunistic parasitic infections in HIV/AIDS patients presenting with diarrhoea by the level of immunesuppression. Indian J Med Res, 130(1):63–6. 19700803

[21] Lono AR Kumar S Chye TT (2008). Incidence of microsporidia in cancer patients. J Gastrointest Cancer, 39(1–4):124–129. 19459072 10.1007/s12029-009-9065-z

[22] Maggi P Larocca AM Quarto M (2000). Effect of antiretroviral therapy on cryptosporidiosis and microsporidiosis in patients infected with human immunodeficiency virus type 1. Eur J Clin Microbiol Infect Dis, 19(3):213–217. 10795595 10.1007/s100960050461

[23] Manatsathit S Tansupasawasdikul S Wanachiwanawin D (1996). Causes of chronic diarrhea in patients with AIDS in Thailand: a prospective clinical and microbiological study. J Gastroenterol, 31(4):533–537. 8844474 10.1007/BF02355053

[24] Matos O Lobo ML Xiao L (2012). Epidemiology of Enterocytozoon bieneusi infection in humans. J Parasitol Res, 2012:981424. 10.1155/2012/981424 PMC3469256 23091702

[25] Missaye A Dagnew M Alemu A Alemu A (2013). Prevalence of intestinal parasites and associated risk factors among HIV/AIDS patients with pre-ART and on-ART attending dessie hospital ART clinic, Northeast Ethiopia. AIDS Res Ther, 10(1):7. 23442332 10.1186/1742-6405-10-7 PMC3598834

[26] Mohandas K Sehgal R Sud A Malla N (2002). Prevalence of intestinal parasitic pathogens in HIV-seropositive individuals in Northern India. Jpn J Infect Dis, 55(3):83–84. 12195048

[27] Nsagha DS Njunda AL Assob NJC (2016). Intestinal parasitic infections in relation to CD4+ T cell counts and diarrhea in HIV/AIDS patients with or without antiretroviral therapy in Cameroon. BMC Infect Dis, 11;16:9. 26754404 10.1186/s12879-016-1337-1 PMC4707727

[28] Ojuromi OT Izquierdo F Fenoy S (2012). Identification and characterization of microsporidia from fecal samples of HIV-positive patients from Lagos, Nigeria. PLoS One, 7(4):e35239. 22496910 10.1371/journal.pone.0035239 PMC3322150

[29] Orlandi PA Lampel KA (2000). Extraction-free, filter-based template preparation for rapid and sensitive PCR detection of pathogenic parasitic protozoa. J Clin Microbiol, 38(6):2271–2277. 10834988 10.1128/jcm.38.6.2271-2277.2000 PMC86779

[30] Pape JW Verdier R-I Boncy M (1994). Cyclospora infection in adults infected with HIV: clinical manifestations, treatment, and prophylaxis. Ann Intern Med, 121(9):654–657. 7944073 10.7326/0003-4819-121-9-199411010-00004

[31] Polley SD Boadi S Watson J Curry A Chiodini PL (2011). Detection and species identification of microsporidial infections using SYBR Green real-time PCR. J Med Microbiol, 60(Pt 4):459–466. 21183599 10.1099/jmm.0.026781-0

[32] Rinder H Thomschke A Dengjel B (2000). Close genotypic relationship between Enterocytozoon bieneusi from humans and pigs and first detection in cattle. J Parasitol, 86(1):185–188. 10701590 10.1645/0022-3395(2000)086[0185:CGRBEB]2.0.CO;2

[33] Ryan NJ Sutherland G Coughlan K B (1993). A new trichrome-blue stain for detection of microsporidial species in urine, stool, and nasopharyngeal specimens. J Clin Microbiol, 31(12):3264–3269. 7508457 10.1128/jcm.31.12.3264-3269.1993 PMC266395

[34] Sak B Brady D Pelikánová M (2011). Unapparent microsporidial infection among immunocompetent humans in the Czech Republic. J Clin Microbiol, 49(3):1064–1070. 21191056 10.1128/JCM.01147-10 PMC3067711

[35] Saksirisampant W Prownebon J Saksirisampant P S (2009). Intestinal parasitic infections: prevalences in HIV/AIDS patients in a Thai AIDS-care centre. Ann Trop Med Parasitol, 103(7):573–581. 19825279 10.1179/000349809X12502035776072

[36] Samie A Obi C Tzipori S Weiss L Guerrant R (2007). Microsporidiosis in South Africa: PCR detection in stool samples of HIV-positive and HIV-negative individuals and school children in Vhembe district, Limpopo Province. Trans R Soc Trop Med Hyg, 101(6):547–554. 17412378 10.1016/j.trstmh.2007.02.005 PMC3109624

[37] Santín M Trout JM Vecino JAC Dubey J Fayer R (2006). Cryptosporidium, Giardia and Enterocytozoon bieneusi in cats from Bogota (Colombia) and genotyping of isolates. Vet Parasitol, 141(3–4):334–339. 16860480 10.1016/j.vetpar.2006.06.004

[38] Sarfati C Bourgeois A Menotti J (2006). Prevalence of intestinal parasites including microsporidia in human immunodeficiency virus–infected adults in Cameroon: a cross-sectional study. Am J Trop Med Hyg, 74(1):162–164. 16407362

[39] Sobottka I Schwartz DA Schottelius J (1998). Prevalence and clinical significance of intestinal microsporidiosis in human immunodeficiency virus-infected patients with and without diarrhea in Germany: a prospective coprodiagnostic study. Clin Infect Dis, 26(2):475–480. 9502473 10.1086/516328

[40] Sokolova OI Demyanov AV Bowers LC (2011). Emerging microsporidian infections in Russian HIV-infected patients. J Clin Microbiol, 49:2102–2108. 21450962 10.1128/JCM.02624-10 PMC3122743

[41] Sulaiman Im Bern C Gilman R (2003). A Molecular Biologic Study of Enterocytozoon bieneusi in HIV-Infected Patients in Lima, Peru. J Eukaryot Microbiol, 50:591–596. 14736175 10.1111/j.1550-7408.2003.tb00642.x

[42] Van Gool T Snijders F Reiss P (1993). Diagnosis of intestinal and disseminated microsporidial infections in patients with HIV by a new rapid fluorescence technique. J Clin Pathol, 46(8):694–699. 8408691 10.1136/jcp.46.8.694 PMC501450

[43] Van Hal S Muthiah K Matthews G (2007). Declining incidence of intestinal microsporidiosis and reduction in AIDS-related mortality following introduction of HAART in Sydney, Australia. Trans R Soc Trop Med Hyg, 101(11):1096–100. 17662322 10.1016/j.trstmh.2007.06.003

[44] Velasquez J Carnevale S Cabrera M (2004). [Cyclospora cayetanensis in patients with AIDS and chronic diarrhea]. Acta Gastroenterol Latinoam, 34(3):133–137. 15742928

[45] Weber R Ledergerber B Zbinden R (1999). Enteric infections and diarrhea in human immunodeficiency virus–infected persons: prospective community-based cohort study. Arch Intern Med, 159(13):1473–1480. 10399899 10.1001/archinte.159.13.1473

[46] Yazar S Yalcln S Sahin Í (2004). Human cyclosporiosis in Turkey. World J Gastroenterol, 10:1844. 15188522 10.3748/wjg.v10.i12.1844 PMC4572285

[47] Zali MR Mehr AJ Rezaian M (2004). Prevalence of intestinal parasitic pathogens among HIV-positive individuals in Iran. Jpn J Infect Dis, 57(6):268–70. 15623953

